# Saline Systems highlights for 2005

**DOI:** 10.1186/1746-1448-2-1

**Published:** 2006-01-17

**Authors:** Shiladitya DasSarma

**Affiliations:** 1 University of Maryland Biotechnology Institute, Center of Marine Biotechnology, Baltimore, Maryland 21202, USA

## Abstract

On the 4^th ^of July, 2005, the Saline Systems editorial group launched the new online open access journal, *Saline Systems*, with BioMed Central as the publisher. The scope of the journal includes both basic and applied research on halophilic organisms and saline environments, from gene systems to ecosystems. The stated goal of the journal is to meet publication needs for researchers working in coastal and inland saline environments and provide an interdisciplinary and readily accessible forum for scientists worldwide. The inaugural volume of the journal contains a significant number of high quality original research papers and reviews on a wide range of relevant topics. At the end of the launch period, from January 1, 2006 onwards, the journal will be introducing article-processing charges to cover the cost of publication. Charges will be partly or completely waived for authors from BioMed Central institutional subscribers and in cases of financial hardship.

## Research papers

The first volume of *Saline Systems *[1] included research papers from genomics and proteomics to physiology, ecology, and limnology. On the molecular end of the spectrum, a paper by McCready et al. [2] provided an insightful DNA microarray study on the effects of UV radiation on gene expression in a model extreme halophile, which is highly resistant to solar radiation. Results indicated that genes for homologous recombination are induced by UV. Another molecular study was published by Kan et al. [3] on the environmental proteomics of Chesapeake Bay, Maryland, USA. This study demonstrated the power of metaproteomics to link taxonomic diversity, functional diversity, and biological processes in natural environments.

In one microbial ecology paper, Echigo et al. [4] surveyed the greater Tokyo area of Japan for halophilic bacteria, from the seashore to inland regions. Although samples collected from the seashore yielded much smaller total numbers of bacteria than those of inland soil samples, the numbers of halophilic bacteria per gram were nearly the same. These results suggested that halophiles may be dispersed via dust storms originating in deserts in the interior Asia. In another ecological study, Major et al. [5] published the first in a series designed to catalog and characterize the Salt Plains National Wildlife Refuge ecosystem in Oklahoma, USA. Despite the harsh and highly variable conditions observed in this environment, a higher than expected phototrophic species diversity was observed.

Two research papers relevant to aquaculture reported studies on shrimp species, *Branchipus schaefferi *and *Artemia franciscana. *Sarma et al. [6] reported that both survival and offspring production of *B. schaefferi *are reduced at higher salinity levels, suggesting that this species is unlikely to be successful in colonizing inland saline water bodies and that proper conservation measures must be taken for their protection. Camargo et al. [7] reported studies on *A. franciscana *occurring along the Colombian Caribbean coast, focusing on the species' quality for marine larvaeculture and aquaculture.

## Literature reviews

Three reviews appeared in the first volume on diverse aspects of saline systems. A historical review was provided by Oren [8] on halophilic algal species of *Dunaliella*, some of which can tolerate a wide range of salinity. This paper was a retrospective to mark the centennial of the discovery of these intriguing halophiles. Second, an extensive review of the organic compatible solutes of halotolerant and halophilic microorganisms was published by Roberts [9]. This review included both biosynthetic pathway information and the properties of compatible solutes that effect molecular stability. Third, a comprehensive review of saline lakes in the Great Plains region of western Canada authored by Last and Ginn [10] presented hydrochemical analysis of 800 lakes.

## Book and meeting reviews

A review of a new treatise on halophilic microorganisms was published in volume 1 of *Saline Systems *by Rensing [11]. This book entitled "Adaptation to life at high salt concentrations in Archaea, Bacteria, and Eukarya" collected the proceedings of a triennial conference, "Halophiles", which was held in Ljubljana, Slovenia, in September 2004. Finally, reflections on the 9th International Conference on Salt Lake Research held in Perth, Australia in September 2005 were published in a commentary by Jellison [12].

## Article-processing charges

An important footnote is that our publisher, BioMed Central, is introducing article-processing charges (APCs) from January 1, 2006 onwards to recover the cost of publication. APCs are essential for the sustainability of open access publishing in general and for the success of *Saline Systems *in particular. Authors should note that open access, made economically viable through APCs, provides many advantages over the traditional subscription model with its restriction to access and dissemination of knowledge (authors know that the existence of subscriptions does not exclude page and/or color charges). In the open access model, authors continue to retain copyright ownership to their publications and do not have to be concerned about the length of their manuscript or the number of figures, since the APC is a flat charge. The use of color does not incur any additional charges, and the articles are archived in perpetuity in the US NIH's PubMed Central and other archives and repositories, with free access over the world-wide-web.

Authors affiliated with institutional members of BioMed Central, of which there are over 500 at present, will continue to enjoy the advantages of membership. In many cases this will be a full waiver of APCs, although in some cases it will be a partial waiver, depending on the membership type. For those authors with hardship cases, individual waiver requests will be considered and may be granted in cases of lack of funds. A waiver application is required during the submission process, and a decision on the waiver will normally be made within two working days.

## Epilog

The past year was an exciting, fruitful, and successful inaugural year for *Saline Systems*. In 2006, we invite submissions of high quality manuscripts on all aspects of halophiles and saline environments.
